# Presenilins in Alzheimer’s disease and frontotemporal dementia

**DOI:** 10.1186/1750-1326-7-S1-L8

**Published:** 2012-02-07

**Authors:** Jie Shen

**Affiliations:** 1 Center for Neurologic Diseases, Program in Neuroscience, Harvard Medical School, Boston, Massachusetts, USA

## Background

Synaptic dysfunction is widely thought to be an important pathogenic event in Alzheimer’s disease (AD). Presenilins, which harbor large numbers of mutations for familial AD and frontotemporal dementia (FTD), are important for neurotransmitter release and synaptic plasticity as well as memory and age-related neuronal survival.

## Results

Our recent report showed that presenilins regulate synaptic function by modulating ryanodine receptor-mediated calcium release from the ER. To determine how presenilins regulate intracellular calcium signaling in neurons, we performed Ca^2+^ imaging coupled with electrophysiological and molecular analyses using both acute hippocampal slices of unique *presenilin* conditional mutant mice and cultured hippocampal neurons, in which presenilins are acutely inactivated with a lentivirus expressing Cre recombinase.

## Conclusion

Our results reveal a selective interaction between presenilins and ryanodine receptors in the regulation of calcium homeostasis and synaptic function, and suggest that disruption of calcium homeostasis may be an early pathogenic event leading to synaptic dysfunction in AD. We also generated two *presenilin-1* knockin mice, in which either an AD- (L435F) or FTD (G183V) -causing mutation is introduced into the respective *presenilin-1* endogenous genomic locus, to investigate the mechanisms by which presenilin mutations cause AD or FTD. The analysis of these mutant mice will also be presented.

